# Injectable Thermosensitive Chitosan/Pullulan-Based Hydrogels with Improved Mechanical Properties and Swelling Capacity

**DOI:** 10.3390/polym12112514

**Published:** 2020-10-28

**Authors:** Prakasit Panyamao, Warintorn Ruksiriwanich, Panee Sirisa-ard, Suporn Charumanee

**Affiliations:** 1 Department of Pharmaceutical Sciences, Chiang Mai University, Chiang Mai 50200, Thailand; prakasit.panyamao@gmail.com (P.P.); warintorn.ruksiri@cmu.ac.th (W.R.); pmpti008@gmail.com (P.S.-a.); 2 Cluster of Research and Development of Pharmaceutical and Natural Products Innovation for Human or Animal, Chiang Mai University, Chiang Mai 50200, Thailand

**Keywords:** chitosan, genipin, β-Glycerophosphate, hydrogels, pullulan, thermosensitive

## Abstract

Thermosensitive chitosan/β-glycerophosphate (CS/BGP) systems have been developed as injectable hydrogels. However, the hydrogels exhibited poor mechanical properties due to their physically crosslinked networks. In this work, CS/BGP hydrogels were reinforced by covalent crosslinking using genipin (GE) and concomitantly semi-interpenetrating networks using pullulan (PL). Based on response surface methodology, the optimized formulation was composed of CS (1.05%, *w/v*), PL (1%, *w/v*), BGP (6%, *w/v*), and GE (70.79 mcg/mL). The optimized hydrogels exhibited Young’s modulus of 92.65 ± 4.13 kPa and a percentage of equilibrium swelling ratio of 3259.09% ± 58.90%. Scanning electron micrographs revealed a highly porous structure with nanofibrous networks in the CS/PL/BGP/GE hydrogels. The chemical interactions between the compositions were investigated by Fourier-transform infrared spectroscopy. Rheological measurements illustrated that the optimized hydrogels displayed sol–gel transition within one minute at 37 °C, a lower critical solution temperature of about 31 °C, and viscoelastic behavior with high storage modulus. Furthermore, the optimized hydrogels demonstrated higher resistance to in vitro enzymatic degradation, compared to the hydrogels without GE. Our findings could suggest that the thermosensitive CS/PL/BGP/GE hydrogels with enhanced mechanical properties and swelling capacity demonstrate the potential for use as scaffolds and carriers for cartilage tissue engineering and drug delivery applications.

## 1. Introduction

Hydrogels are biomaterials described as three-dimensional networks of hydrophilic polymers constructed via either physical or chemical crosslinking. As a result of the formation of interconnected networks, the hydrogels present highly porous structures that allow the ability to absorb a large amount of water or biological fluids [1,2]. Moreover, such highly porous architecture provides the hydrogels high swelling capacity and permeability of oxygen, nutrients, wastes, and water-soluble molecules. The desired properties of hydrogels for biomedical applications are biocompatibility and similarity to the natural extracellular matrix (ECM) of soft tissues, as well as sufficient mechanical strength [3,4,5].

Over the past few decades, injectable hydrogels or in situ-forming hydrogels have received more attention in biomedical applications than other smart biomaterials. The advantages of injectable hydrogels are easy administration in liquid form, minimally invasive surgery, the possibility to fill up irregularly shaped sites, and allowing simple incorporation of bioactive molecules [6,7]. Generally, injectable hydrogels are developed by utilizing thermo-responsive polymers having lower critical solution temperature (LCST) behavior. In principle, LCST polymers undergo sol-to-gel transition with increasing temperature above their specific LCST point due to the alteration in the hydrophilic/hydrophobic balance within the hydrogel networks. In the case of in situ injectable hydrogels, the polymers should exhibit an LCST point below physiological temperature [8,9,10,11].

Chitosan (CS) is a linear polycationic polysaccharide. It is derived from partial deacetylation of chitin, which is extracted from the exoskeleton of crustaceans, fungi, and insects [12]. The chemical structure of CS is composed of D-glucosamine and *N*-acetyl-D-glucosamine linked via β-(1,4) glycosidic linkage [13]. CS is soluble in dilute acidic medium (pH < 6.3), since its free amino groups are protonated, causing the electrostatic repulsions between its chains [14]. Interestingly, CS becomes a thermosensitive polymer upon mixing with polyol-phosphate salts, such as β-glycerophosphate (BGP) [15]. The CS/BGP systems display a clear solution at room temperature, even though their pH is increased above 6.3, but transform into a gel state whenever the temperature is elevated near 37 °C [16,17]. Based on the intrinsic properties, CS exhibits excellent biocompatibility, biodegradability, low immunogenicity, non-cytotoxicity, antimicrobial activities, mucoadhesive properties, and its structural similarity to glycosaminoglycan found in cartilage [13,18,19].

Thermosensitive hydrogels based on CS/BGP systems are widely used in cartilage regeneration because they possess good injectability, ability to fulfil the cartilage defects, and rapid gelation at the injection sites [17,19]. However, the previous studies reported that CS/BGP hydrogels exhibited poor mechanical properties and a high degradation rate in physiological conditions due to their physically crosslinked networks, thus limiting their applications [20,21,22]. To overcome these drawbacks, covalent crosslinking together with double networks are applied [23,24,25]. Genipin (GE), a non-cytotoxic crosslinking agent, is extracted from gardenia fruits. GE acts as a dialdehyde that reacts spontaneously with primary amine groups of CS chains through the Schiff-base reaction to form covalently crosslinked networks [15,26,27]. Previous studies demonstrated that the mechanical properties of CS-based hydrogels were enhanced by the crosslinking with GE [20,28,29,30,31]. However, a high degree of crosslinking causes a decrease in swelling capacity of the hydrogels owing to the restriction of the water diffusion [32]. Pullulan (PL) is a linear non-ionic polysaccharide produced by *Aureobasidium pullulans*. It is an edible polysaccharide with biocompatible, biodegradable, and no cytotoxic properties [33,34]. The addition of PL into CS solutions provides high flexibility for the resultant materials due to the formation of semi-interpenetrating networks (semi-IPNs) [35,36].

In this work, we aimed to improve mechanical properties, as well as swelling capacity, for injectable thermosensitive hydrogels based on CS/BGP systems intended for use as scaffolds for cartilage tissue engineering. The hydrogels were prepared by crosslinking CS with GE and blending with PL to form semi-IPN hydrogels. The effects of the formulation compositions on Young’s modulus (*E*) and percentage of equilibrium swelling ratio (%ESR) were investigated using a Box–Behnken design with response surface methodology, and the optimal formulation was evaluated. Furthermore, the properties of optimized hydrogels, including gelation time, injectability, morphology, FTIR spectra, rheological properties, and in vitro biodegradation, were characterized.

## 2. Materials and Methods

### 2.1. Materials

Chitosan (MW: 250 kDa, degree of deacetylation: ≥ 90%) was purchased from Marine Bio Resources (Samut Sakhon, Thailand). Pullulan (low viscosity, oligosaccharides: ≤5 wt %) was purchased from Chanjao Longevity (Bangkok, Thailand). Genipin (≥ 98%) and β-glycerophosphate disodium salt hydrate (L-α-isomer: ≤ 1.0 mol %) and lysozyme from chicken egg white (enzyme activity: 70,000 U/mg) were purchased from Sigma-Aldrich (St. Louis, MO, USA). All other chemical reagents were of analytical grade and were used without further purification.

### 2.2. Preparation of Thermosensitive CS/PL-Based Hydrogels

Thermosensitive CS/PL-based hydrogels were prepared by physical blending. Briefly, CS (1.75 g) was dissolved in 100 mL of acetic acid (0.1 mol/L) under magnetic stirring at room temperature for 3 h, while various concentrations of PL solutions were separately prepared in deionized water. Then, PL solutions were added into a CS solution with a volume ratio of 1:3. The resulting solutions were kept in a refrigerator before use. Afterwards, 1 mL of the precooled BGP solutions with varying concentrations were slowly dropped onto the CS/PL blends (4 mL) under gentle magnetic stirring and cooling in an ice-bath, followed by 50 μL of different GE concentrations. The mixtures were continuously stirred for 10 min prior to characterization. The prepared formulations were transparent and homogeneous solutions. Each composition was optimized to accomplish the optimal formulation, based on the maximum mechanical properties and highest swelling capacity.

### 2.3. Experimental Design

A Box–Behnken design (BBD) is defined as a three-level fractional factorial with an incomplete block design used to statistically evaluate the interactions between independent variables. A BBD is also described as a spherical response surface methodology (RSM) that consists of a central point and the middle points of the edges of the cube without any points at the vertices [37,38]. In this study, the final concentration of CS was assigned to be constant at 1.05% (*w/v*), while the concentrations of PL (*X*_1_), BGP (*X*_2_), and GE (*X*_3_) were the independent variables. The sets of experiments were generated by Design-Expert software (Version 10.0). The selected factors and their levels are presented in Table 1. The effects of the three independent variables on the two critical responses (*Y*_1_: *E* and *Y*_2_: %ESR) were investigated using RSM. The optimal formulation was obtained based on the desirability, which was analyzed from the software. The second-order polynomial model suggested by RSM was explained by the following equation:

(1) $$\;Y\;=\;\beta_{0}\;+\;\sum \beta_{i}X_{i}\;+\;\sum \beta_{ii}X_{i}^{2}\;+\;\sum \beta_{ij}X_{i}X_{j}\;+\;e,$$

where *Y* is the predicted response; the terms of $\beta_{0}$, $\beta_{i}$, $\beta_{ii}$, and $\beta_{ij}$ (*i =* 1, 2, 3; *j* = 1, 2, 3; *i* < *j*) represent the regression coefficients for intercept, linear, quadratic, and interactions, respectively; $X_{i}$ and $X_{j}$ are the coded independent variables [39,40].

### 2.4. Determination of Gelation Time

Sol–gel transition for each of the hydrogels was investigated by the tube inversion method [41,42,43]. Briefly, the vials containing sample solutions were initially incubated in a water bath with a controlled temperature of 37 °C for 30 s and then vertically inverted every 15 s. The gelation time was considered whenever a solid-like sample was obtained. Each sampling procedure was performed in triplicate.

### 2.5. Syringeability

Syringeability is assessed as the force required to inject sample solutions [44,45]. The measurement of the injection force was performed using a TA-XT Plus texture analyzer (Stable Micro Systems, Surrey, UK) in a compression mode at room temperature. A 3-mL syringe, containing the freshly prepared sample (1 mL) was positioned via vertical support at the holder. Then, a stainless-steel cylindrical probe with a 25 mm diameter (P/25) was pressed onto the plunger end of the syringe with a crosshead test speed of 1 mm/s and a target distance of 10 mm in order to flow the samples through a 22-gauge needle. The maximum injection force was evaluated from a texture analysis profile. All the experiments were conducted in triplicate.

### 2.6. Mechanical Tests

The mechanical properties of the hydrogels were evaluated by indentation method [46]. The mechanical properties of all experimental runs were examined using a TA-XT Plus texture analyzer in a compression mode at room temperature. The Delrin hemispherical probe (P/0.5HS) with a 0.5-inch diameter was compressed onto the surface of the formed hydrogels with a minimum trigger force of 5 g and a test speed of 0.5 mm/s. Then, the probe was detached from the sample when the target displacement reached 2 mm. All samples were analyzed for three separate tests. The maximum compression force and indentation distance were obtained from the texture analysis profile. *E* of the hydrogels was then calculated using Hertz’s equation for spherical indentation [46,47,48], described by the following equation:

(2) $$F\;=\;\frac{4ER^{\frac{1}{2}}\delta^{\frac{3}{2}}}{3\left(1\;-\nu^{2}\right)}$$

where *E* and *F* represent Young’s modulus and compression force, respectively; *R* and *δ* are radius of the indenter and indentation depth, respectively; *ν* is Poisson’s ratio (an incompressible material like hydrogel had Poisson’s ratio of 0.5) [46,49,50,51].

### 2.7. Swelling Experiments

Swelling capacity of the hydrogels was measured by gravimetric method [20]. The formed hydrogels were immersed in 15 mL of phosphate-buffered saline (PBS) (pH 7.4) at 37 °C for 24 h to ensure that all 16 hydrogels reached their equilibrium swelling states. After definite time intervals, the hydrogels were gently wiped with a filter paper to remove the excess PBS on their surface and weighed as the swollen weight (*W_s_*) using an Explorer EX125D Semi-Micro balance (Ohaus, Parsippany, NJ, USA). All the swollen hydrogels were then lyophilized at −90 °C and a vacuum condition of 0.1 mbar for 48 h using a Beta 2-8 LD-plus freeze-dryer (Christ, Osterode am Harz, Germany). The freeze-dried hydrogels were weighed again to obtain the dry weight (*W_d_*). All sampling procedures were performed in triplicate. The percentage of equilibrium swelling ratio (%ESR) was calculated as the following equation:

(3) $$\mathrm{ESR}\;\left(\%\right)\;=\;\frac{W_{s}{\;–\;W}_{d}}{W_{d}}\;\times \;100.$$

### 2.8. Morphological Characterization

The morphology of the hydrogels was investigated by scanning electron microscopy (SEM) analysis. The lyophilized hydrogel was cross-sectioned, directly mounted onto stubs, and sputter-coated with 30 nm-thick gold. The samples were observed using a JSM-5410LV scanning electron microscope (JEOL, Tokyo, Japan) at an accelerating voltage of 10 kV.

### 2.9. Fourier-Transform Infrared Spectroscopy

Fourier-transform infrared (FTIR) spectra of the raw materials and the lyophilized hydrogels were characterized using a Nicolet 6700 FTIR spectrometer (Thermo Scientific, Madison, WI, USA) equipped with a single-bounce diamond crystal of attenuated total reflectance (ATR) accessory. All IR spectra were recorded with 32 scans at a 4.0 cm^−1^ resolution in the wavelength ranges between 4000 and 650 cm^−1^.

### 2.10. Rheological Measurements

The oscillatory rheological experiments of the optimized hydrogels were performed using a HAAKE MARS III rheometer (Thermo Scientific, Karlsruhe, Germany) with a 35 mm diameter cone (2° angle) and a plate rheometer equipped with a Peltier unit to control the temperature [52]. The desired gap between cone and plate was set to 0.105 mm. To identify the linear viscoelastic (LVE) region, the strain sweep experiments were carried out from 0.10% to 1000% strain amplitude at a constant frequency of 1 Hz and 37 °C. The frequency sweeps were conducted in a frequency ranging from 0.1 to 100 Hz within the LVE range (10% deformation). The gelation time was determined by time sweeps at a fixed temperature of 37 °C with a frequency of 1 Hz and 10% strain, while the gelation temperature was investigated by increasing temperature from 25 to 40 °C at a heating rate of 1 °C/min. The storage modulus (G’) and the loss modulus (G’’) were recorded.

### 2.11. In Vitro Enzymatic Degradation

Biodegradation of the optimized hydrogels was studied by incubating the hydrogels in an enzymatic solution and then measured their weight loss [20]. The formed hydrogel (1 g) was soaked in 15 mL of PBS (pH 7.4) at 37 °C for 3 h at which the three hydrogels (CS/PL/BGP, CS/PL/GE, and CS/PL/BGP/GE) reached their equilibrium swelling states. Afterwards, the excess PBS was carefully eliminated, and the initial weight (*W_i_*) of hydrogels was measured. Then, 15 mL of the enzymatic solution (0.1% (*w/v*) lysozyme in PBS (pH 7.4)) was added into the swollen hydrogels and further incubated at 37 °C for specific times of 1, 2, 4, 7, 14, 21, 28, 35, and 42 days. At each period, the hydrogel was recovered from the medium, and re-weighed to obtain the final weight (*W_t_*). The enzymatic degradation was evaluated from the percentage of weight loss, calculated using the according equation:

(4) $$\mathrm{Weight}\;\mathrm{loss}\;\left(\%\right)\;=\;\frac{W_{i}{\;–\;W}_{t}}{W_{i}}\;\times \;100.$$

### 2.12. Statistical Analysis

All data were reported as means ± standard deviation (SD). The multiple regression analysis and the polynomial equations for each response were obtained from the design software. Statistical analyses were performed using analysis of variance (ANOVA). The level of significant difference was defined as *p* < 0.05.

## 3. Results and Discussion

### 3.1. Sol–Gel Transition of CS/PL-Based Hydrogels

**Figure 1:**
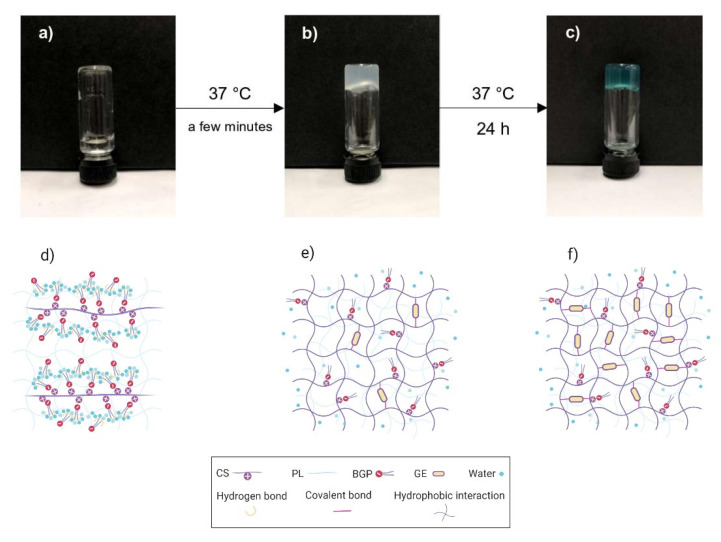
Schematic illustration of the sol-to-gel transition of chitosan (CS)/pullulan (PL)/β-glycerophosphate (BGP)/genipin (GE) hydrogels. The freshly prepared solution (**a**,**d**), physically crosslinked hydrogels (**b**,**e**), and co-crosslinked hydrogels (**c**,**f**).

Figure 1 schematically illustrates the sol–gel transition of the CS/PL/BGP/GE hydrogels, obtained from the tube inversion tests, and an anticipated crosslinking mechanism in hydrogel networks. The freshly prepared sample behaved like a clear solution with low viscosity at room temperature (Figure 1a,d). It suddenly stopped flowing and turned into a turbid gel after incubation at 37 °C for a few minutes (Figure 1b,e). This indicated the transformation from a polymer solution to a physically crosslinked gel. When the formed hydrogels were continuously incubated up to 24 h, it could be seen that the turbid gels turned into blue-colored hydrogels (Figure 1c,f). As expected, the results in Figure 1 revealed that CS-based hydrogels exhibited sol–gel transition at body temperature after BGP was added. An explanation is that an elevated temperature weakened hydrogen-bonding interactions between polyol groups of BGP and water molecules. This meant that the hydrophobic attractions of CS chains were dominant, resulting in promotion of the gel formation [16,17,53]. Moreover, the addition of GE caused the formation of blue pigments of the hydrogels, indicating that GE molecules could effectively crosslink to amino groups of CS chains [26,30,32]. The tube inversion tests demonstrated that the sol–gel transitions of all 16 formulations were observed within 5 min at 37 °C (Figure S1). The syringeability test, evaluated from texture analysis profiles, showed that all the experimental runs required the maximum injection force in the range of 7 to 15 N to expel the prepared solutions from a syringe. The injection forces of all samples were below the upper limit of a reasonable injection force of 30 N [54]. However, it was noted that the injection force was elevated with increased PL concentration due to higher viscosity (Figure S2).

### 3.2. Morphological Analysis

**Figure 2:**
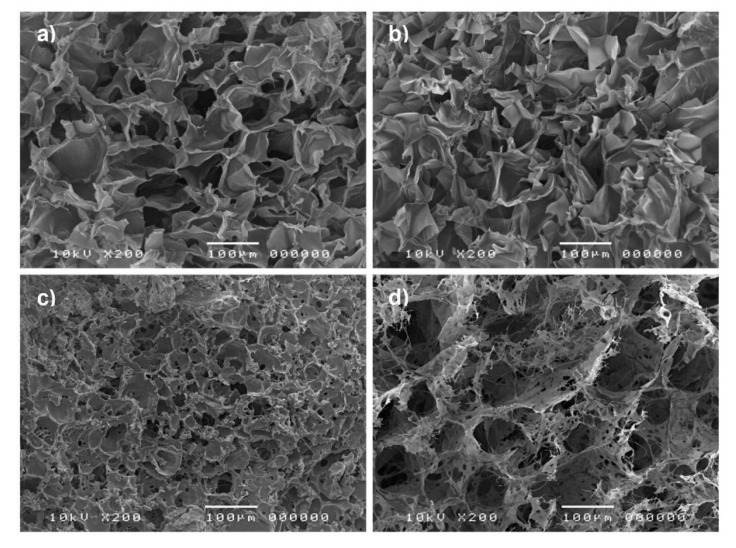
SEM images of pure CS (**a**), CS/PL (**b**), CS/PL/BGP hydrogels (**c**), and CS/PL/BGP/GE hydrogels (**d**).

SEM images revealed the surface morphology and porous nature of the freeze-dried CS/PL-based hydrogels, as shown in Figure 2. The image for pure CS showed that it possessed a macroporous structure and smooth pore walls with a large surface area (Figure 2a), and its morphology was similar to CS/PL blends (Figure 2b). Porous structure with various pore sizes ranging from several microns to around 50 µm at the surface of hydrogels was observed in CS/PL/BGP hydrogels (Figure 2c). Interestingly, the internal structure of CS/PL/BGP hydrogels crosslinked with GE showed a highly porous structure in irregular patterns, and their pores were interconnected with nanofibrous structures (Figure 2d). It was suggested that the crosslinking of CS with GE created the micro- and nano-scale pores for the hydrogels [29,30].

### 3.3. ATR-FTIR Analysis

**Figure 3:**
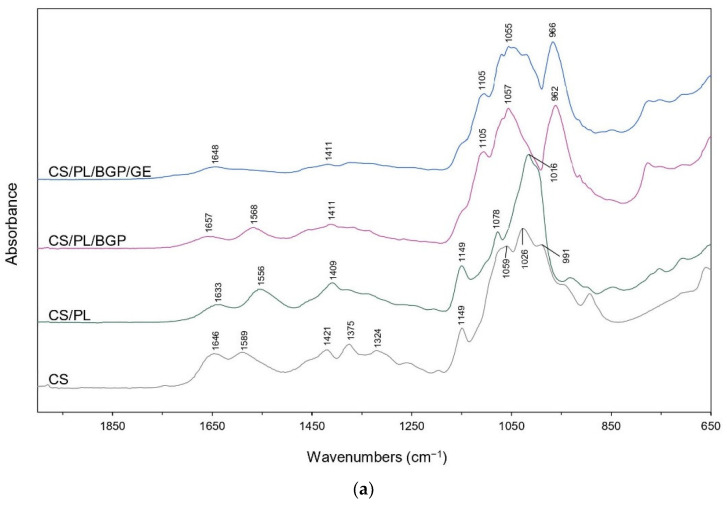

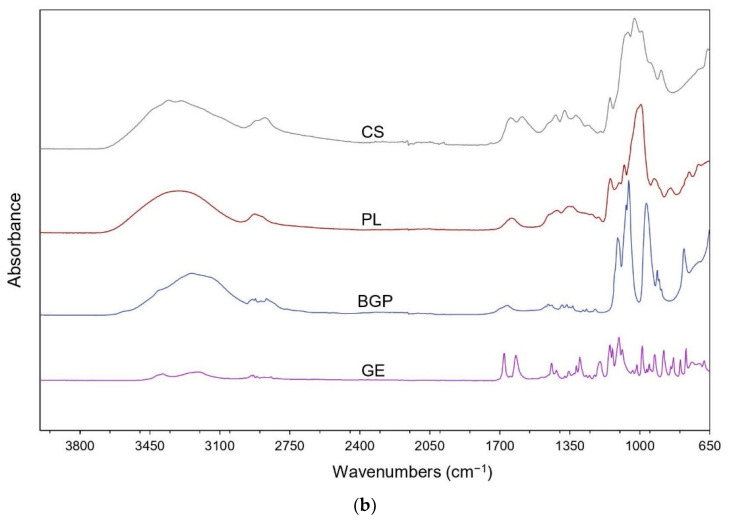
Attenuated total reflectance-Fourier-transform infrared (ATR-FTIR) spectra of CS/PL-based hydrogels (**a**) and raw materials (**b**).

ATR-FTIR spectra of the prepared CS/PL-based hydrogels together with their raw materials are shown in Figure 3. In the spectrum of pure CS, a strong broad band in the region of 3600 to 3000 cm^−1^ represented the stretching vibrations of the OH and NH groups. The bands at 2924 and 2876 cm^−1^ were assigned to asymmetric and symmetric C–H stretching in the pyranose ring of CS, while the vibrations of CH_2_ bending and CH_3_ symmetrical bending were observed at 1421 and 1375 cm^−1^, respectively [55,56]. The bands located at 1646, 1589, and 1325 cm^−1^ corresponded to the C=O stretching in amide I, N–H bending of primary amine groups, and C–N stretching in amide III, respectively. These bands indicated that CS was the partially deacetylated product of chitin [57,58]. Frequency shifts to lower wavenumbers of amide I (from 1646 to 1633 cm^−1^) and primary amine peaks (from 1589 to 1556 cm^−1^) were observed in the mixed CS/PL. This might be associated with the hydrogen-bonding interactions between hydroxyl groups of PL between amino groups of CS [35,36,59]. Nevertheless, the addition of BGP into CS/PL led to the shifts of the C=O stretching and the N–H bending to higher frequencies at 1657 and 1568 cm^−1^, respectively. This was due to the ionic interactions between the protonated amino groups (−${\mathrm{NH}}_{3}^{+}$) of CS and phosphate groups (–${\mathrm{PO}}_{4}^{3–}$) of BGP [20,60,61]. In the case of CS/PL/BGP/GE hydrogels, their IR spectral characteristics were almost similar to those of CS/PL/BGP hydrogels. However, the peak for N–H bending vibration disappeared. An explanation is that the free amino groups of CS chains were consumed for crosslinking with GE to form a secondary amide [31,62].

### 3.4. Experimental Design and Statistical Analysis

Sixteen experimental runs were generated based on the BBD using Design-Expert software. The design matrix together with the experimental and predicted responses are shown in Table 2. On the basis of multiple regression analysis, the mathematical models for describing the relationship between the three independent variables in the coded levels and the responses are shown by the following second-order polynomial equations:

(5) $$Y_{1}\;=\;71.25\;+\;{3.13X}_{1}\;–\;{11.23X}_{2}\;+\;{7.73X}_{3}\;–\;{2.83X}_{1}X_{2}\;–\;{3.37X}_{1}X_{3}\;+\;{0.27X}_{2}X_{3}\;+\;{6.19X}_{1}^{2}\;–\;{0.40X}_{2}^{2}\;+\;{3.19X}_{3}^{2},$$

(6) $$Y_{2}\;=\;2881.79\;–\;{329.83X}_{1}\;–\;{97.70X}_{2}\;–\;{34.16X}_{3}\;+\;{45.31X}_{1}X_{2}\;–\;{13.46X}_{1}X_{3}\;–\;{13.58X}_{2}X_{3}\;+\;{10.61X}_{1}^{2}\;\;–\;{42.51X}_{2}^{2}\;+\;{36.82X}_{3}^{2},$$

where *Y*_1_ and *Y*_2_ represent the predicted responses of *E* and %ESR, respectively; *X*_1_, *X*_2_, and *X*_3_ are the coded variables in the concentration of PL, BGP, and GE, respectively.

The reliability of the proposed models was further statistically analyzed through ANOVA. The ANOVA results for *E* (*Y*_1_) and %ESR (*Y*_2_) are given in Tables S1 and S2, respectively. It could be seen that the model *F*-values of *Y*_1_ and *Y*_2_ were 177.09 and 132.83, respectively, with very small probability values (*p* < 0.0001 for both *Y*_1_ and *Y*_2_). This implied that both the regression models were statistically significant. The *F*-values of lack-of-fit (0.56 for *Y*_1_ and 0.61 for *Y*_2_) were not significant relative to the pure error. There was a 67.57% (*Y*_1_) and 65.36% (*Y*_2_) chance that “lack of fit *F*-value” could occur due to noise. These results could suggest that the quadratic models fitted well with the experimental data.

Regarding the significance of each coefficient term, if the *p*-value was less than 0.05, the coefficient could be considered as the significant term. It could be seen that all the linear terms (*X*_1_, *X*_2_, and *X*_3_), two interaction terms (*X*_1_*X*_2_ and *X*_1_*X*_3_), and two quadratic terms ($X_{1}^{2}$ and $X_{3}^{2}$) for *E* were significant, while the other terms were not significant. In the case of %ESR, the terms of *X*_1_, *X*_2_, *X*_3_, *X*_1_*X*_2_, $X_{2}^{2}$, and $X_{3}^{2}$ were significant coefficients, but the others were insignificant. In addition, the two models provided high values of the adjusted determination coefficient (adj-*R*^2^ = 0.9906 for *Y*_1_ and 0.9875 for *Y*_2_). This indicated that 99.06% and 98.75% of the total variations on both *E* and %ESR could be explained by the fitted models. These adj-*R*^2^ values were in reasonable agreement with the pred-*R*^2^ values (0.9741 for $Y_{1}$ and 0.9643 for $Y_{2}$). This suggests that the experimental values and predicted responses were well correlated.

### 3.5. Effect of Formulation Compositions on the Responses

The graphical representations of the regression equations are shown in the two-dimensional (2D) contour and three-dimensional (3D) response surface plots, which were employed to investigate the interaction effects between the independent variables on each response. The experimental data were plotted with a function of two variables by fixing the other variable at its zero level [37,38,39,40].

**Figure 4:**
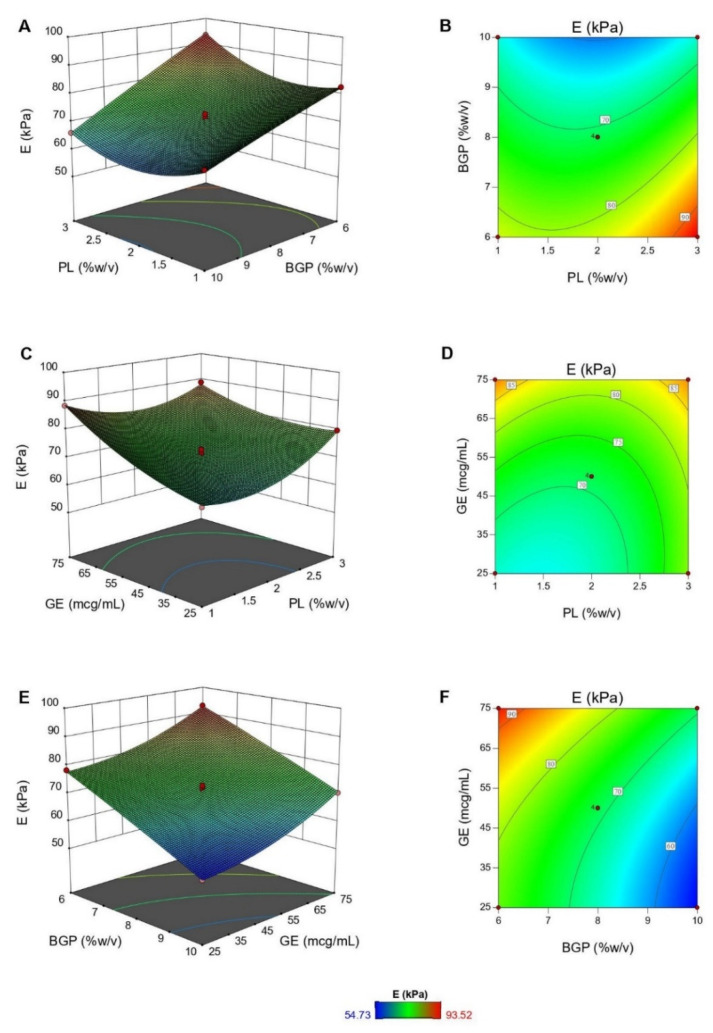
The response surface plots showing the interaction effects of PL and BGP (**A**), PL and GE (**C**), and BGP and GE (**E**) on Young’s modulus (*E*), together with the corresponding contour plots (**B**,**D**,**F**).

Figure 4 illustrates the 3D response surface and 2D contour plots for the interaction effects between formulation compositions (PL, BGP, and GE concentrations) on Young’s modulus. Based on ANOVA results, although all the three variables significantly influenced *E* of hydrogels, the significant interaction effects were *X*_1_*X*_2_ and *X*_1_*X*_3_ terms. *E* decreased dramatically with an increase in BGP concentration at any PL content. For example, at high PL concentration (3%, *w/v*), *E* declined from 93.52 to 65.96 kPa as BGP concentration rose between 6% and 10% (*w/v*) (Figure 4A,B). Likewise, when BGP concentration was fixed at medium level, it was shown that increased PL or GE concentration also stiffened the hydrogels (Figure 4C,D). This suggests that the crosslinked networks of CS are also reinforced by the formation of semi-IPNs with a linear polymer, like PL [35,36,63]. Furthermore, a larger amount of GE increased the crosslinking degree of the networks and thus enhanced mechanical properties of the hydrogels [28,29,30,31]. However, the covalent crosslinking reaction, a slow process, was hindered by the ionic interactions when the BGP concentration was high. Consequently, the physically crosslinked networks prevailed, leading to a decrease in mechanical properties of hydrogels, as shown in Figure 4E,F [20,64].

**Figure 5:**
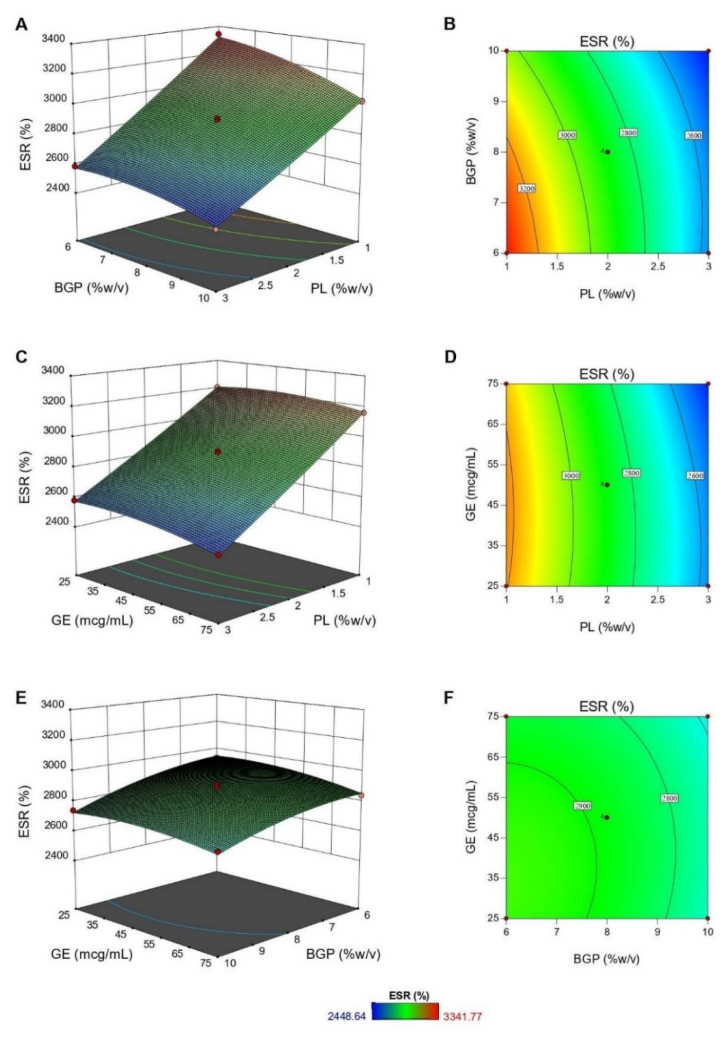
The response surface plots showing the interaction effects of PL and BGP (**A**), PL and GE (**C**), and BGP and GE (**E**) on percentage of equilibrium swelling ratio (%ESR), together with the corresponding contour plots (**B**,**D**,**F**).

Figure 5 demonstrates the plots for the relationship between independent variables and %ESR. According to the results of ANOVA, while all independent variables significantly affected %ESR of hydrogels, only the interaction term of *X*_1_*X*_2_ was significant. It was clear that %ESR of the hydrogels markedly declined as the PL concentration rose from 1% to 3% (*w/v*) (Figure 5A–D) and slightly decreased upon the increase of BGP and GE concentrations (Figure 5E,F). Such a reduction was due to the formation of a rigid network at a higher concentration of crosslinking agents and PL [20,31]. An increase in PL contents caused an increase in intermolecular hydrogen bonds between PL and CS chains that tightened the hydrogel networks and reduced the void spaces available for water absorption [36]. Similarly, a higher concentration of BGP reduced the electrostatic repulsion between CS chains and thus led to a drop in swelling ability [64,65].

### 3.6. Verification of the Predictive Models

The desired formulation of thermosensitive CS/PL-based hydrogels should exhibit the maximum *E* and %ESR. According to multi-response optimization, the optimal formulation was obtained based on the desirability, which ranged between 0 and 1. The high value of desirability indicated the stable variability of the response [66]. In this study, the highest desirability was 0.958 (Figure S3) and the corresponding formulation was composed of 1.05% (*w/v*) CS, 1% (*w/v*) PL, 6% (*w/v*) BGP, and 70.79 mcg/mL GE. The predicted values of *E* and %ESR were 93.52 kPa and 3268.78%, respectively. The optimized hydrogels were further characterized in triplicate to validate the prediction for practical use. From the results of experiments, *E* and %ESR of the hydrogels were 92.65 ± 4.13 kPa and 3259.09% ± 58.90%, respectively. The data showed that the experimental data were very close to the predicted values of the regression models.

The optimized hydrogels showed higher *E* than CS/BGP hydrogels, as shown in Figure S4. Moreover, the previous studies reported that the mechanical properties in term of the elastic modulus of CS/BGP hydrogels were 1.5 kPa [22], 3.95 kPa [67], 6.65 kPa [68], and 0.5–1 kPa [69], which were lower than that of the optimized hydrogels. For the swelling capacity, the optimized hydrogels showed greater %ESR than CS/BGP hydrogels reported in the literature, such as 850% [31], 923% [67], 880% [68], and 2814% [69].

### 3.7. Rheological Characterization

**Figure 6:**
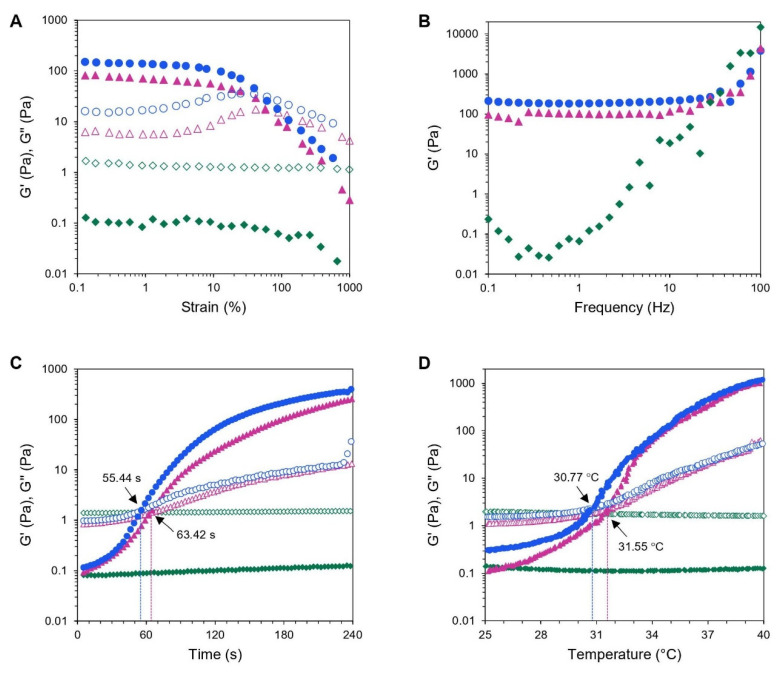
Rheological measurements of CS/PL-based hydrogels: amplitude strain sweeps (**A**), frequency sweeps (**B**), time sweeps (**C**), and temperature sweeps (**D**). Storage moduli and loss moduli (G’, G’’) of CS/PL (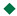,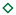), CS/PL/BGP (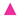, 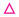), and CS/PL/BGP/GE (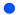, 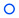).

Hydrogels are described viscoelastic materials that are deformed under very small strain without destroying their structure [70]. To study the dynamic rheological properties of hydrogels, oscillatory strain-sweep tests are employed to identify a linear viscoelastic (LVE) region, where the storage modulus (G’) and loss modulus (G”) are independent of the applied strain up to a critical deformation [71,72]. In this study, the strain sweep tests were carried out from 0.1% to 1000% strain amplitude at a constant frequency of 1 Hz and 37 °C on the fully formed hydrogels. Figure 6A illustrates that both CS/PL/BGP and CS/PL/BGP/GE hydrogels had G’ values greater than their G’’ values, reflecting that an elastic gel had constructed. In addition, it could be observed that the LVE regions for both hydrogels ranged between 0.1% and 10% of the applied strain. This meant that the hydrogel networks were destructed when the strain was greater than 10%. However, the CS/PL sample without crosslinking agents showed a liquid-like behavior (G’ < G’’) throughout the strain range.

After the LVE region was defined, a frequency sweep test was performed at a strain below the critical strain to characterize the formation of hydrogel networks that reflect to the elastic modulus of hydrogels [70,72]. The frequency-dependence analysis of G’ was conducted in the frequency range between 0.1 and 100 Hz within the LVE region. As shown in Figure 6B, it could be seen that G’ values for CS/PL/BGP and CS/PL/BGP/GE hydrogels were independent from the frequency below 30 Hz but showed a certain dependency at higher frequency, whereas G’ for CS/PL elevated continuously as a function of frequency increased. As expected, dual crosslinking provided stronger hydrogels with large G’ values [21,65,73,74]. This was in agreement with the results of mechanical testing obtained from a texture analyzer.

The sol–gel transition the point at which G’ crosses G” is determined as a function of time or temperature, corresponding to loss of flowability of hydrogels owing to abrupt change in viscoelastic properties from an initially liquid-like to a solid-like behavior [72]. According to Figure 6C, the time sweeps at 37 °C revealed that no crossover point between G’ and G” was detected in the physically mixed CS/PL samples, since both polymers were not intrinsically thermo-responsive. Nevertheless, the CS/PL blends could exhibit thermosensitive properties with the presence of BGP, which is in accordance with the literature [21,58,73]. The gelation of CS/PL/BGP occurred at 63.42 s, and this decreased to 55.44 s upon the addition of GE. It was suggested that the chemical crosslinking of GE could accelerate the gelation process of the CS/BGP system [20,31,65]. Hence, the optimized hydrogels could presumably transform into a gel within a few minutes after being administered into the body.

Dynamic temperature ramp studies were performed to characterize thermally induced sol–gel transition of hydrogels by increasing temperature in the range of 25 to 40 °C. The temperature sweep curves demonstrated that no sol–gel transition was observed in CS/PL hydrogel without crosslinking agent, whereas the gelation temperature of CS/PL/BGP was at 31.55 °C and decreased to 30.77 °C after the addition of GE (Figure 6D). The results indicated that GE facilitated gel formation by covalently crosslinking [73]. Even if the optimized hydrogels exhibited a gelation temperature below body temperature, i.e., 30.77 °C, the hydrogels remained a viscous sol at controlled room temperature.

### 3.8. In Vitro Enzymatic Degradation

**Figure 7:**
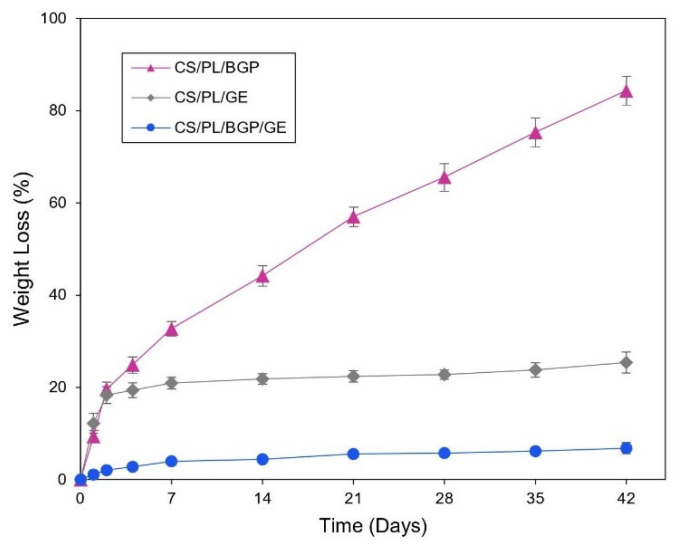
In vitro biodegradation profiles of CS/PL-based hydrogels prepared with BGP (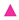), GE (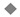), and both BGP and GE (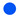).

The degradation of the hydrogel networks can undergo via many mechanisms, such as erosion, solubilization, and hydrolysis [75]. Lysozyme is an enzyme that hydrolyzes the β-(1,4) glycosidic linkage of CS backbone causing degradation of the hydrogels [76]. In this study, lysozyme was added into PBS medium in which all samples were immersed in order to mimic erosive in vivo environments. The biodegradation rate of the optimized CS/PL-based hydrogels was monitored by the percentage of weight loss with time after incubated with an enzymatic solution. Figure 7 illustrates that the rate of weight loss for the CS/PL/BGP hydrogels was increased dramatically to hit over 80% after incubation for 42 days. Nevertheless, the CS/PL/GE hydrogels rapidly deteriorated in the first two days and then slowly degraded to reach 25% of the original mass at the end of the study. As expected, it was found that the optimized hydrogels (CS/PL/BGP/GE) had very slow degradation, approximately 6% of their initial mass during the incubation period. These results were similar to the previous reports [20,21,31] that the chemical crosslinking of CS chains via GE was much more resistant to enzymatic degradation than the physical crosslinking by BGP. The co-crosslinked hydrogels were more durable to lysozyme due to the restriction of enzyme penetration into the polymer chains, which subsequently retarded the cleavage of glycoside linkage.

## 4. Conclusions

In this study, injectable thermosensitive CS/PL-based hydrogels were prepared by simple mixing. A Box–Behnken experimental design (BBD) with response surface methodology (RSM) was effectively employed to investigate the effects of three selected compositions on Young’s modulus (*E*) and percentage of equilibrium swelling ratio (%ESR) and to predict the optimal formulation. According to RSM, *E* was enhanced with an increase in PL and GE concentrations, while it was markedly decreased as the amount of BGP increased. The %ESR was significantly decreased upon a rise in PL concentration, whereas a slight reduction of %ESR was observed upon the increase of BGP and GE concentrations. The optimized hydrogels, which exhibited the highest *E* and %ESR, consisted of 1.05% (*w/v*) CS, 1% (*w/v*) PL, 6% (*w/v*) BGP, and 70.79 mcg/mL GE. This hydrogel demonstrated a highly porous structure with nanofibrous interconnected pores. The rheological studies revealed that the optimized formulation formed a strong hydrogel within 1 min at 37 °C, and its LCST point was about 31 °C. Furthermore, the optimized hydrogels showed higher resistance to enzymatic degradation. Our findings revealed that the thermosensitive CS/PL/BGP/GE hydrogels can rapidly transform into a rigid gel at a physiological temperature with satisfactory mechanical properties and swelling capacity. These hydrogels show promising potential for use as carriers or scaffolds for drug delivery and cartilage tissue engineering applications.

## Figures and Tables

**Table 1:** Independent variables and their levels in the Box–Behnken design (BBD).

Independent Variables		Levels	
	Low (−1)	Medium (0)	High (+1)
*X*_1_: PL concentration (%, *w/v*)	1	2	3
*X*_2_: BGP concentration (%, *w/v*)	6	8	10
*X*_3_: GE concentration (mcg/mL)	25	50	75

**Table 2:** Design matrix for the three coded factors with the experimental and predicted responses.

Runs	Factors			Responses			
	*X*_1_	*X*_2_	*X*_3_	*E* (kPa)		ESR (%)	
				Experimental	Predicted	Experimental	Predicted
F1	−1	−1	0	82.45 ± 5.19	82.31	3341.77 ± 32.62	3322.74
F2	+1	0	+1	88.76 ± 1.14	88.12	2482.10 ± 55.04	2478.13
F3	−1	0	−1	65.76 ± 5.36	66.40	3202.14 ± 15.68	3206.11
F4	0	0	0	72.85 ± 2.14	71.25	2908.54 ± 68.62	2881.79
F5	+1	−1	0	93.52 ± 4.97	94.23	2582.54 ± 27.65	2572.46
F6	0	+1	+1	70.30 ± 1.35	70.80	2699.23 ± 10.70	2684.18
F7	+1	0	−1	79.62 ± 3.02	79.40	2578.34 ± 49.14	2573.37
F8	0	0	0	70.26 ± 4.61	71.25	2894.69 ± 13.46	2881.79
F9	0	0	0	71.53 ± 3.19	71.25	2835.63 ± 100.92	2881.79
F10	0	+1	−1	54.73 ± 1.77	54.80	2739.41 ± 62.41	2725.35
F11	0	0	0	70.36 ± 3.71	71.25	2888.30 ± 37.31	2881.79
F12	−1	+1	0	66.23 ± 5.16	65.51	3026.64 ± 78.90	3036.72
F13	−1	0	+1	88.38 ± 1.48	88.60	3159.75 ± 66.13	3164.72
F14	+1	+1	0	65.96 ± 4.98	66.10	2448.64 ± 22.52	2467.67
F15	0	−1	−1	78.30 ± 4.61	77.80	2932.85 ± 44.76	2947.90
F16	0	−1	+1	92.80 ± 6.80	92.72	2838.37 ± 12.95	2852.43

*X*_1_, *X*_2_, and *X*_3_ represent the concentration of PL, BGP, and GE, respectively. Experimental data are reported as mean ± SD (*n* = 3).

## Supplementary Materials

The following are available online at https://www.mdpi.com/2073-4360/12/11/2514/s1, Figure S1: The gelation time of the prepared CS/PL/BGP/GE hydrogels based on Box–Behnken design, Figure S2: The injection force of the prepared CS/PL/BGP/GE hydrogels based on Box–Behnken design, Figure S3: Desirability of the optimized formulation, Figure S4: Comparison of the mechanical properties between CS/BGP and the optimized CS/PL/BGP/GE hydrogels, Table S1: Results of ANOVA for Young’s modulus (*E*), Table S2: Results of ANOVA for percentage of equilibrium swelling ratio (%ESR).
