# Supplementary material for: Injectable Thermosensitive Chitosan/Pullulan-Based Hydrogels with Improved Mechanical Properties and Swelling Capacity
Source: Polymers (Basel). 2020 Oct 28;12(11):2514. doi: 10.3390/polym12112514 (PMC7692642; doi:10.3390/polym12112514)
Supplement: Supplementary file 1 [file polymers-12-02514-s001.pdf]

polymers

MDPI

# Injectable Thermosensitive Chitosan/Pullulan-Based Hydrogels with Improved Mechanical Properties and Swelling Capacity

**Prakasit Panyamao [1], Warintorn Ruksiriwanich [1,2], Panee Sirisa-ard [1], Suporn Charumanee [1,*]**

[1] Department of Pharmaceutical Sciences, Chiang Mai University, Chiang Mai 50200, Thailand; prakasit.panyamao@gmail.com (P.P.), warintorn.ruksiri@cmu.ac.th.com (W.R.), pmpti008@gmail.com (P.S.-a.)

[2] Cluster of Research and Development of Pharmaceutical and Natural Products Innovation for Human or Animal, Chiang Mai University, Chiang Mai 50200, Thailand

* Correspondence: chsuporn@gmail.com; Tel.: +66-82-694-3996

Received: date; Accepted: date; Published: date

## Supplementary Materials

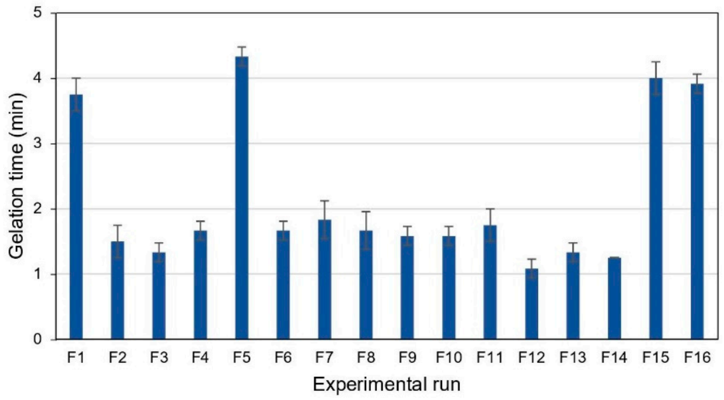


**Figure 1.** The gelation time of the prepared chitosan (CS)/pullulan (PL)/β-glycerophosphate (BGP)/genipin (GE) hydrogels based on Box-Behnken design (BBD).

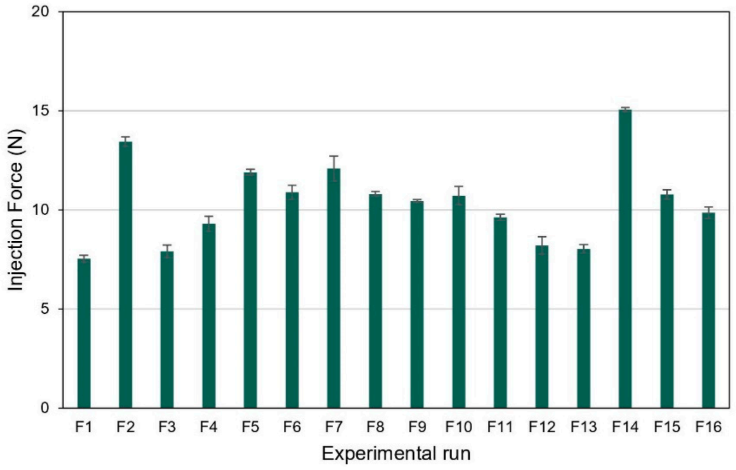


**Figure 2.** The injection force of the prepared CS/PL/BGP/GE hydrogels based on BBD.

**Table 1.** Results of ANOVA for Young's modulus (*E*).

| **$Y_1$: Young's modulus; *E*** | | | | | | |
|---|---|---|---|---|---|---|
| **Source** | **Sum of Squares** | **Degree of Freedom** | **Mean Square** | ***F*-Value** | ***p*-value (Prob > *F*)** | **Remarks** |
| Model | 1837.93 | 9 | 204.21 | 177.09 | < 0.0001 | significant |
| $X_1$ | 78.36 | 1 | 78.36 | 67.95 | 0.0002 | significant |
| $X_2$ | 1009.18 | 1 | 1009.18 | 875.16 | < 0.0001 | significant |
| $X_3$ | 477.89 | 1 | 477.89 | 414.43 | < 0.0001 | significant |
| $X_1X_2$ | 32.09 | 1 | 32.09 | 27.83 | 0.0019 | significant |
| $X_1X_3$ | 45.47 | 1 | 45.47 | 39.43 | 0.0008 | significant |
| $X_2X_3$ | 0.29 | 1 | 0.29 | 0.25 | 0.6345 | not significant |
| $X_1^2$ | 153.39 | 1 | 153.39 | 133.02 | < 0.0001 | significant |
| $X_2^2$ | 0.65 | 1 | 0.65 | 0.57 | 0.4807 | not significant |
| $X_3^2$ | 40.62 | 1 | 40.62 | 35.22 | 0.0010 | significant |
| Residual | 6.92 | 6 | 1.15 | – | – | |
| Lack of fit | 2.49 | 3 | 0.83 | 0.56 | 0.6757 | not significant |
| Pure error | 4.43 | 3 | 1.48 | – | – | |
| Correlated total | 1844.85 | 15 | – | – | – | |
| Adj-$R^2$ = 0.9906, Pred-$R^2$ = 0.9741, CV = 1.42% | | | | | | |

*$X_1$*, *$X_2$* and *$X_3$* represent the concentration of PL, BGP, and GE, respectively.

**Table 2.** Results of ANOVA for percentage of equilibrium swelling ratio (%ESR).

| *Y*2: %ESR | | | | | | |
|---|---|---|---|---|---|---|
| **Source** | **Sum of Squares** | **Degree of Freedom** | **Mean Square** | ***F*-Value** | ***p*-value (Prob > *F*)** | **Remarks** |
| Model | 978792.78 | 9 | 108754.75 | 132.83 | < 0.0001 | significant |
| $X_1$ | 870319.34 | 1 | 870319.34 | 1063.00 | < 0.0001 | significant |
| $X_2$ | 76364.33 | 1 | 76364.33 | 93.27 | < 0.0001 | significant |
| $X_3$ | 9335.09 | 1 | 9335.09 | 11.40 | 0.0149 | significant |
| $X_1X_2$ | 8211.25 | 1 | 8211.25 | 10.03 | 0.0194 | significant |
| $X_1X_3$ | 724.89 | 1 | 724.89 | 0.89 | 0.3831 | not significant |
| $X_2X_3$ | 737.17 | 1 | 737.17 | 0.90 | 0.3793 | not significant |
| $X_1^2$ | 450.43 | 1 | 450.43 | 0.55 | 0.4863 | not significant |
| $X_2^2$ | 7227.05 | 1 | 7227.05 | 8.83 | 0.0249 | significant |
| $X_3^2$ | 5423.24 | 1 | 5423.24 | 6.62 | 0.0421 | significant |
| Residual | 4912.48 | 6 | 818.75 | – | – | |
| Lack of fit | 1857.44 | 3 | 619.15 | 0.61 | 0.6536 | not significant |
| Pure error | 3055.04 | 3 | 1018.35 | – | – | |
| Correlated total | 983705.26 | 15 | – | – | – | |
| Adj-$R^2$ = 0.9875, Pred-$R^2$ = 0.9643, CV = 1.00% | | | | | | |

$X_1$, $X_2$ and $X_3$ represent the concentration of PL, BGP, and GE, respectively.

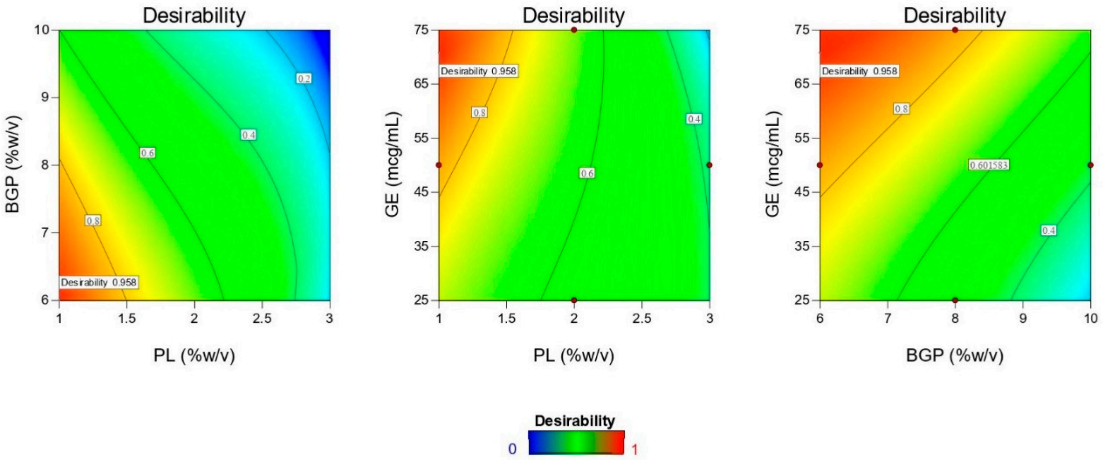


**Figure 3.** Desirability of the optimized formulation.

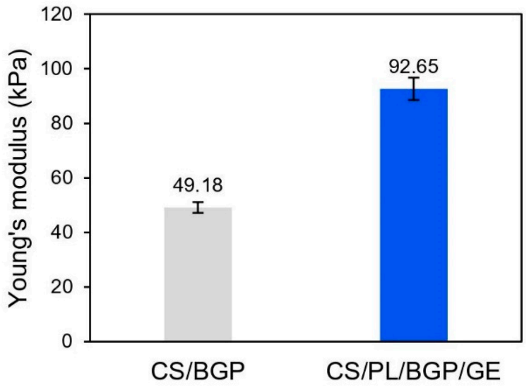


**Figure 4.** Comparison of the mechanical properties between CS/BGP and the optimized CS/PL/BGP/GE hydrogels.

© 2020 by the authors. Licensee MDPI, Basel, Switzerland. This article is an open access article distributed under the terms and conditions of the Creative Commons Attribution (CC BY) license (http://creativecommons.org/licenses/by/4.0/).
